# Unsupervised Classification of Local Clathrate Hydrate Structures

**DOI:** 10.1021/acs.jpcc.5c07202

**Published:** 2026-02-16

**Authors:** Xinrui Cai, Alberto Striolo, Matteo Salvalaglio

**Affiliations:** † Department of Chemical Engineering, 4919 University College London, London WC1E 7JE, United Kingdom; ‡ School of Sustainable Chemical, Biological and Materials Engineering and Materials Science and Engineering Program, 522128 University of Oklahoma, Norman, Oklahoma 73019, United States

## Abstract

Quantifying and differentiating the structural characteristics of clathrate hydrates at the molecular level is crucial for understanding the properties that underpin hydrate-based technologies. While useful, current approaches lack sufficient resolution to discern, e.g., interfacial and dynamical structures. In this study, we present an algorithm based on Density-Based Spatial Clustering of Applications with Noise (DBSCAN) that accurately identifies different water states coexisting within clathrate hydrates. A key novel component is an effective cavity-finder algorithm, which provides input to the clustering framework. The new algorithm detects hydrate cavities by analyzing the number and type of constituent molecular rings around voids. Integrating the new algorithm with widely used order parameters (e.g., *F*3, *F*4, and *F*4*t*) provides a powerful and accurate tool for analyzing hydrate structures at interfaces and phase transitions. The performance of the new algorithm is assessed for structure I (sI) CO_2_ hydrates and for structure II (sII) mixed CH_4_/Dioxane hydrates, demonstrating its robustness and adaptability across different clathrates. Crucially, the proposed algorithm enables us to identify partially ordered structures characteristic of the quasi-liquid layer, thereby capturing interfacial dynamics and molecular-scale details essential for understanding hydrates under realistic conditions.

## Graphical abstract

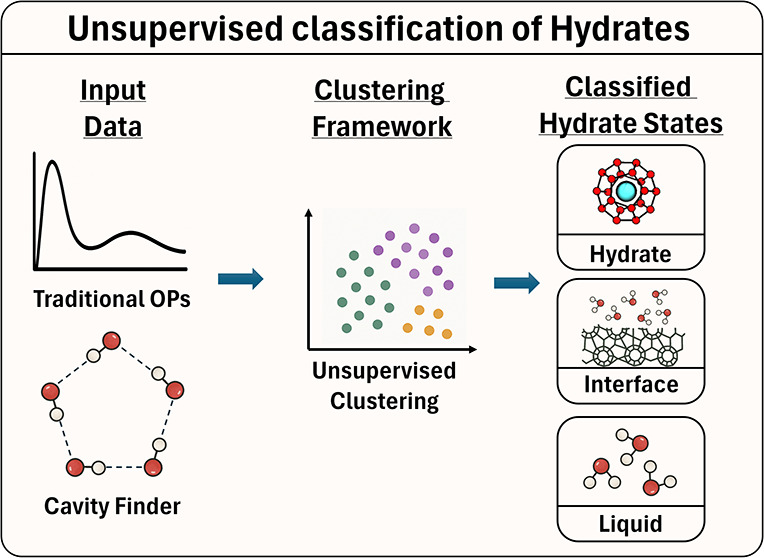

## Introduction

Clathrate hydrates are crystalline compounds formed by water molecules assembled into cage-like ordered three-dimensional structures.^1^ Water–water hydrogen-bonds and dispersive interactions between water and guest molecules such as carbon dioxide and methane, entrapped within hydrate cages, stabilize the crystalline structures, even at pressure–temperature conditions at which ice would not be stable.^2,3^ Fundamentally interesting because of their unique structures, clathrate hydrates are also attracting wide public attention due to their possible application in a variety of cutting-edge technologies, ranging from carbon capture and storage^4–6^ to water desalination,^7,8^ from gas separation^9^ to intermittent natural gas storage.^10,11^ From a more mundane viewpoint, in the energy industry, hydrates can be a nuisance because their formation in oil and gas pipelines can lead to blockages and ruptures.^12,13^ Whether to promote cutting-edge technologies or to prevent adverse effects due to their untimely formation, it is crucial to understand the properties and behavior of hydrates under various scenarios.

Molecular simulations have become an increasingly valuable tool for studying hydrate systems, as they provide critical insights into the underlying driving forces and mechanisms at the microscopic and, when possible, molecular level. These simulations often complement and help interpret experimental observations, enabling a deeper understanding of complex phenomena that are difficult to capture through experiments alone. Indeed, techniques such as molecular dynamics (MD) and Monte Carlo (MC) simulations can resolve at the atomistic scale phenomena such as hydrate nucleation, growth, thermodynamic stability, and phase transitions, enabling a comprehensive understanding of their behavior under various conditions.^14–18^ For these approaches to be effective, it is crucial to identify the local arrangement of water molecules within the hydrate framework and to distinguish, with confidence, different hydrate structures, as well as liquid water, solely from analysis of the simulated trajectories. Several approaches have been proposed for such discrimination, including the quantification of *order parameters*. For example, *F3* is a three-body order parameter that quantifies the deviation of water molecules’ arrangement from a tetrahedral angle.^19^ In contrast, *F4* is a four-body order parameter^19^ frequently used for hydrate structural identification. Vatamanu et al. proposed distinguishing hydrates from liquid water using the displacement of water molecules.^20^ These order parameters (F3, F4, displacement) are global descriptors that rely on spatially averaged values to distinguish among states within a simulated system. They can be used to identify interfacial layers by locating the peak of their normalized derivatives.^21^ While their implementation is usually simple, their effectiveness in capturing local structural variations is unfortunately limited in some cases.^22^ To improve resolution, more complicated algorithms such as GRADE have been developed. GRADE uses the connectivity between water molecules to determine 5^12^, 5^12^6^2^, and 5^12^6^4^ cages.^23^ While accurate for bulk hydrates, this approach comes at a significant computational cost. Machine learning methods have also been developed to identify water structures. Takahashi et al., e.g., employed supervised machine learning to distinguish between ice Ih, sI hydrates, and sII hydrates.^24^ Nevertheless, because they rely on extensive data sets, these algorithms face challenges in identifying interfacial layers and hydrate cages of less common structures, such as dimethyl ether hydrate with its 4^1^5^10^6^3^ structure.^25^

Although the algorithms discussed above have proven helpful in the literature, distinguishing between solid-like, liquid-like, and interfacial local structures remains a challenging task, particularly in efficiently and accurately identifying these local structures. This gap can compromise investigations aimed at quantifying the mechanisms of hydrate growth under various conditions. For example, to quantify the effect of disorder at the solid/liquid interface on crystal growth, it is imperative to distinguish water molecules constituting hydrate cages from liquid molecules and waters with *in-between* structural and dynamic properties. This difficulty is accentuated when the interfacial disorder gives rise to a quasi-liquid layer (QLL). QLLs, in fact, consist of molecular layers of liquid formed on crystal structures. Particularly important in the context of ice studies,^26,27^ QLLs are expected to substantially influence hydrate growth, polymorphism, decomposition, and mechanical properties.^28–32^

The purpose of this study is to develop a computationally efficient algorithm to distinguish between different hydrate structures, liquid water, and interfacial water structures with sufficiently high accuracy to allow, e.g., the quantification of the QLL thickness and its influence on hydrate growth.^33–35^ To this end, we propose a novel approach to identify molecular rings formed by the connectivity of water molecules around voids, combined with several conventional order parameters and Density-Based Spatial Clustering of Applications with Noise (DBSCAN),^36^ to effectively classify different hydrate phases.

In this manuscript, after describing the new algorithm, we test its computational effectiveness and accuracy by differentiating sI and sII hydrates at the liquid-water interface during growth and dissolution. We document how our approach enables accurate analysis of hydrate structural evolution, differentiating among local environments without the need for spatial or temporal averaging.

## Methodology

In brief, the proposed classification method combines cage identification with order parameters to describe the state of water molecules within a five-dimensional space. DBSCAN is then used to identify highly populated states in this 5D space, yielding robust, unsupervised classification of bulk hydrate, interface, and liquid states of water. In the following, we discuss in detail the order parameters, the cage identification algorithm, the clustering procedure, and their interplay in the classification process.

### Order Parameters

In Figure 1, we reproduce a schematic representing five water molecules organized in a ring, such as those typically found in clathrate hydrates. The figure illustrates the formation of hydrogen bonds between the water molecules and provides a helpful visual for describing the order parameters.

**Figure 1:**
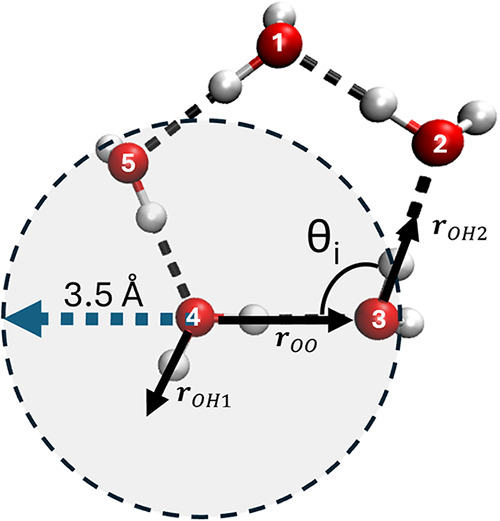
Schematic figure of a typical 5-membered ring formed by water molecules within a hydrate framework. The red spheres represent oxygen atoms, and the white spheres represent hydrogen atoms. The dotted lines represent hydrogen bonds. The numbers identify the five water molecules forming the ring. The hydrogen bonds are identified by geometric criteria that set the distance between O and H atoms, as well as the angle θ formed by the water molecules. The vectors **r**_*OH*1_ and **r**_*OH*2_ lie along the OH bonds that are the farthest away from the two water molecules. Whereas **r**_*OO*_ is the vector joining the two oxygen atoms.

The order parameter F3 for molecule *i*, F3_*i*_ is defined as

1 $$F3_{i}=F3_{i}=\left\langle \right|\cos\theta |\cos\theta +\cos^{2}\left(\varphi_{i}\right)\rangle_{i}$$

In eq 1, θ_*i*_ represents the angle between two vectors joining the oxygen atom of molecule *i* with two oxygen atoms of different molecules within its first solvation shell (see, e.g., Figure 1). The size of the first solvation shell is identified by the oxygen–oxygen radial distribution function.^19^ The φ_*i*_ represents the tetrahedral angle of water molecules. The symbol ⟨···⟩_*i*_ indicates an average over all the triplets of oxygen atoms identified within the first solvation shell of the oxygen belonging to molecule *i*.

On the other hand, the order parameter F4 for molecule *i*, F4_*i*_, is defined as

2 $$F4_{i}=\langle \cos3\phi \rangle_{i}$$

where the angle ϕ_*i*_ refers to any H–O···O–H dihedral angle involving the oxygen atom of molecule *i* and another water molecule within its first solvation shell. The hydrogens defining the dihedral angle are those that are farthest apart.^19^ The averaging ⟨···⟩_*i*_ in eqs 2 and 3 has the same meaning as eq 1.

The order parameter F4t is very similar to F4, however, instead of calculating the dihedral angle ϕ, F4t for molecule *i* is obtained as^37^

3 $$F4t_{i}=\langle {\left(r_{OH_{i}}\times r_{\mathrm{OO}}\cdot r_{OH_{j}}\right)}^{2}\rangle_{i}$$

where, similarly to F4_*i*_, the O–H vectors **r**_OH_*i*__ and **r**_OH_*j*__ are those with hydrogens further apart from each other (see Figure 1).

After computing the set of order parameters for each water molecule, local order parameters were obtained by averaging the values within the first coordination shell, defined as a 6 Å cutoff based on radial distribution function (RDF) analysis. These local descriptors were then normalized to ensure consistent scaling across features. To enhance the resolution of interfacial detection, the gradient of each order parameter within the first coordination shell was also calculated along the axis perpendicular to the interface (the *z*-axis in this study).

### Cage Identification Algorithm

The algorithm proposed here, inspired by GRADE,^23^ face-saturated incomplete cage analysis,^38^ and Hydrogen-bond network analysis,^39,40^ was developed to identify different hydrate cavities in a simulated aqueous system.


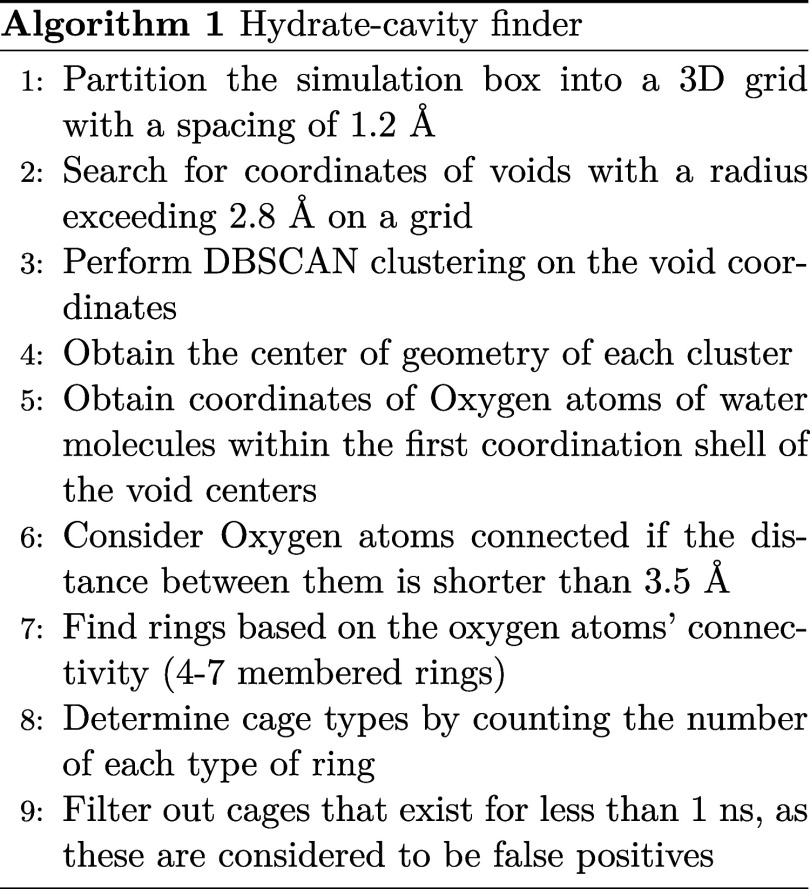


We begin by discretizing the system into a 3D grid with a spacing of 1.2 Å. The algorithm then scans the 3D coordinate space to identify voids with a radius exceeding 2.8 Å (i.e., no water molecules within a 2.8 Å radius). Only water molecules are considered in this search. The criterion of 2.8 Å radius is selected to encompass all hydrate cavities, as the smallest cavities in sI and sII hydrates have radii of 3.95 and 3.91 Å, respectively.^1^ Although the values used here proved effective, grid spacing and void radius can be adjusted based on individual needs.

Since the radius criteria are smaller than the actual radius of hydrate cavities, more than one void coordinate would likely be identified within one hydrate cavity. Therefore, a clustering algorithm is necessary to identify void coordinates that belong to the same hydrate cage and determine the geometrical center of each hydrate cavity. Density-Based Spatial Clustering of Applications with Noise (DBSCAN) is the clustering technique chosen for this step because it does not require knowledge of the number of clusters in each hydrate cage and can deal with uneven cluster sizes.^36^ Once the geometrical center is determined, the algorithm obtains the coordinates of oxygen atoms within a 6 Å radius of the center. This encompasses all water molecules within the first coordination shell of the center of a given hydrate cage.

Once the water molecules in the first coordination shell are identified, a hydrogen-bonded network analysis is performed on the oxygen atoms in these molecules. A common geometric criterion used to identify hydrogen-bonded water molecules is based on the distance between O atoms (r_OO_ < 3.5 Å) and the angle formed by neighboring water molecules (∠HOO < 30°); where r_OO_ is the distance between oxygen atoms and ∠ HOO is the angle between the OH bond and the OO vectors as shown in Figure 1. In the proposed algorithm, however, a contact matrix is used instead of the geometric criterion to search for connectivity between the Oxygen atoms of water molecules. This saves computational power, as we find that there is no need to compute the angles explicitly.

In more detail, using the structure of a typical five-membered ring (Figure 1) as an example, the algorithm first identifies the coordinates of all water molecules within the first coordination shell of the detected void center, as described above. A contact matrix is then constructed, where two water molecules are considered in contact if the distance between their oxygen atoms is less than 3.5 Å. A depth-first search is subsequently performed to determine the connectivity among these molecules. The search begins by identifying a molecule (molecule 2) that is in contact with molecule 1, followed by locating molecule 3, which is in contact with molecule 2 (see numbering shown in Figure 1). The search proceeds sequentially until a fifth molecule is identified. The configuration is classified as a five-membered ring if this fifth molecule, while being in contact with molecule 4, is also connected back to molecule 1. If the fifth molecule does not connect to molecule 1, the algorithm returns to molecule 4 and continues the search for an alternative fifth molecule. Rings of size from four to seven molecules are searched via this algorithm as well. Though not explicitly implemented in the code, rings of larger sized can be identified by adjusting the search parameters according to the algorithm logic.

Because a combination of rings forms hydrate cages, the algorithm can identify different hydrate structures at each time step of the simulation trajectory by counting the number of each ring type at each time interval. Only connected rings that persist for more than 1 ns are considered stable cages in our analysis, which has been found to be a helpful criterion for filtering out false positives. Depending on the needs, shorter or longer-term criteria can be chosen.

### Clustering

The Density-Based Spatial Clustering of Applications with Noise (DBSCAN)^36^ algorithm was employed to classify water molecules into distinct structural states based on the constructed feature set. DBSCAN is a widely used unsupervised clustering method that groups points that are densely packed. Unlike traditional clustering algorithms, DBSCAN does not require prior knowledge of the number of clusters and is particularly effective at identifying clusters of arbitrary shapes, which is advantageous for capturing complex structural variations in hydrate systems. The algorithm operates on two key parameters: the neighborhood radius (ε) and the minimum number of points (MinPts) required to form a dense region.^36^ The neighborhood radius ε was determined by analyzing the distribution of nearest-neighbor distances and selecting a value corresponding to the first significant inflection point in the distribution. Molecules located within of each other and meeting the density requirement are grouped into the same cluster. In this paper, DBSCAN is implemented using the 1.6.1 Sklearn package in Python.^41^

The input to DBSCAN consists of five normalized descriptors: the local order parameters (F3, F4, F4t) and the square of the gradient of F4, along with a binary indicator from the newly developed cavity-finder algorithm. Only the squared gradient of the F4 order parameter was included alongside the other descriptors, as it alone provided sufficient discriminatory power for effective clustering while remaining computationally efficient. Including gradients of additional order parameters did not yield significant improvements in clustering accuracy or precision and was therefore omitted. This framework is also flexible and can accommodate additional order parameters as needed to refine clustering performance.

A sensitivity analysis was performed on the two key DBSCAN parameters, ε and MinPts, and the results are summarized in Figure 3. Overall, variations in these parameters have a negligible impact on the classification of bulk liquid and hydrate molecules, indicating that the algorithm is robust for identifying well-defined phases. In contrast, the identification of interfacial molecules is more sensitive to parameter changes. For example, a 50% decrease in combined with a 20% increase in MinPts can lead to up to a 60% variation in the number of molecules classified as interfacial. This sensitivity at the interface is expected, as interfacial molecules inherently exhibit less-ordered, more heterogeneous local environments than in bulk phases. Small changes in clustering criteria, therefore, primarily affect molecules near the phase boundaries, while leaving bulk classifications largely unchanged. Furthermore, because the interfacial region contains the fewest molecules, parameter variations result in comparatively larger percentage differences. Importantly, this behavior reflects the physical ambiguity in the characterization of interfacial regions rather than a limitation of the algorithm proposed. The stability of liquid and hydrate classifications across a broad parameter range demonstrates that the proposed DBSCAN-based approach provides a reliable and physically meaningful description of hydrate systems.

### Simulation Setup

To test the new algorithm and compare its performance with order-parameter approaches, we conducted molecular dynamics (MD) simulations at atomistic resolution for systems in which liquid water was in contact with a seed hydrate structure. Two different hydrate configurations were considered, representative of systems sI and sII. The former system was used to simulate hydrate growth, while the latter was used to simulate hydrate dissociation. The structure of both sI and sII hydrate cages was built based on the work of Takeuchi.^42^ Periodic boundary conditions were applied in all directions. Both systems were configured such that two hydrate-liquid interfaces are present, aligned perpendicular to the *z*-direction.

The first configuration is arranged as illustrated in Figure 2. The 4 × 4 × 4 sI hydrate slab, fully occupied by 512 CO_2_ molecules, was positioned between two liquid phases. A total of 240 CO_2_ molecules were introduced into the bulk liquid phase, which contained 6,948 water molecules to mimic conditions that favors hydrate growth.^18^ The second configuration was built by 2 × 2 × 2 sII hydrate. 102 Methane and 52 dioxane molecules were positioned within small and large cages of the hydrate seed, respectively. Twenty-six small and 12 large cavities, randomly selected across the hydrate slab, were kept empty. The liquid phase contains 1088 water molecules. No other additives are present (Figure 3).

**Figure 2:**
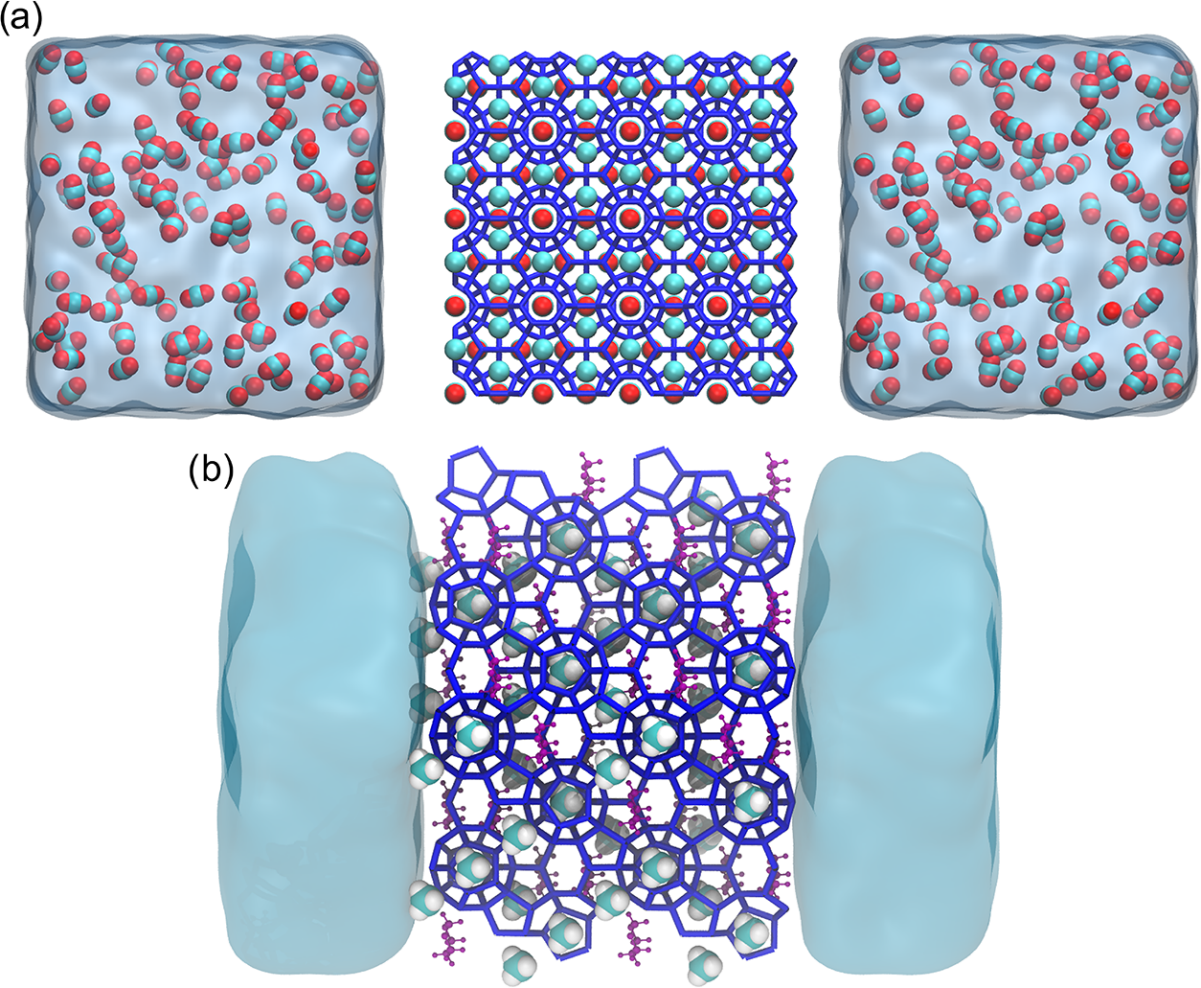
Initial configuration of (a) sI CO_2_ hydrate. The hydrate slab, represented by dark blue lines, lies between two liquid water reservoirs. CO_2_ molecules are present in the hydrate and also in the liquid phase. Carbon and Oxygen atoms of CO_2_ molecules are shown as cyan and red spheres, respectively. Liquid water is shown as a continuous light blue region. (b) sII CH_4_-Dioxane hydrate. CH_4_ molecules occupy the small cages of the hydrate. 1,3-Dioxane molecules occupy the large cage of the hydrates. CH_4_ molecules are represented as cyan and white spheres for Carbon and Hydrogen atoms, respectively. Purple lines and spheres represent 1,3-Dioxane. The liquid is represented in cyan.

**Figure 3:**
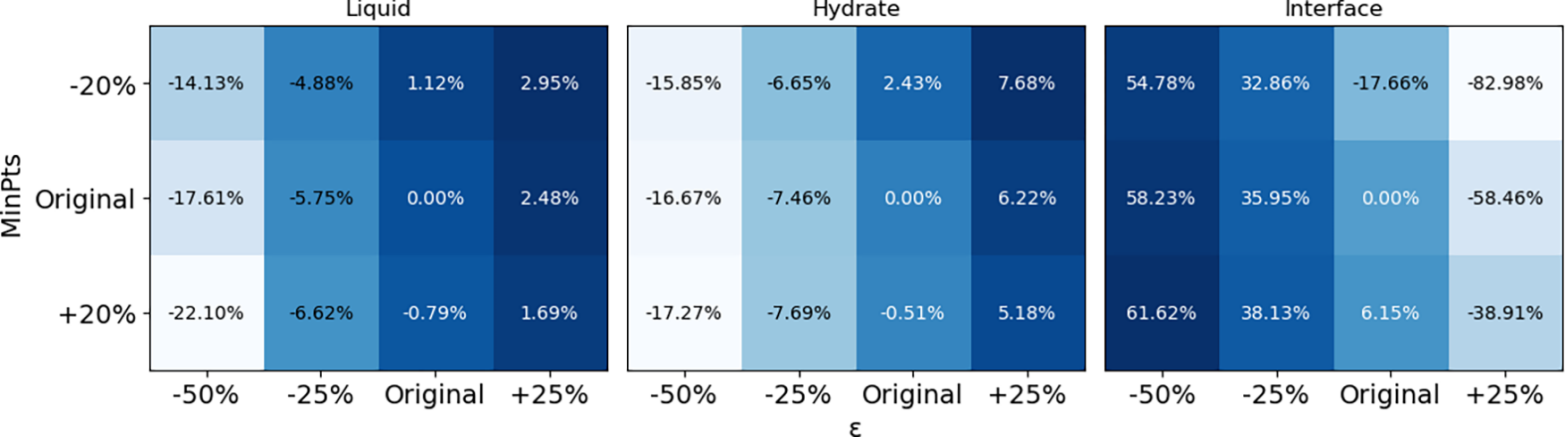
Sensitivity analysis of the two parameters: ε from −50% to +25%, and MinPts from −25% to +25%. The heatmap indicates the percentage change in the number of water molecules classified as bulk liquid, hydrate, and the interface, respectively.

The TIP4P/Ice^43^ and the EPM2 ^44^ force fields were used to describe water and CO_2_ molecules, respectively, as these force fields are reliable in modeling CO_2_ hydrate systems.^15,45,46^ For CH_4_ and Dioxane, the OPLS-AA force field was used instead.^47^ Nonbonded interactions were modeled using electrostatic and dispersion forces. Both electrostatic and dispersion interaction potentials are described with a cutoff of 1.4 nm. The particle-mesh Ewald method was implemented to account for long-range electrostatic effects. Lorentz–Berthelot combining rules were used to estimate dispersive interactions between dissimilar atoms.^48^

The GROMACS 2021 software package was employed to integrate the equations of motion.^49^ The leapfrog algorithm was used to solve the equation of motion with a time step of 1 fs. The initial configuration was energy-minimized using the steepest descent algorithm, followed by relaxation under NVT constraints for 1 ns. The systems were then simulated within the NPT ensemble for 2 ns to achieve equilibration. The Berendsen pressure coupling^50^ with a time constant of 5 ps and the Nosé-Hoover thermostat^51^ with a time constant of 0.5 ps were employed. Finally, NPT simulations were performed for both systems using Nosé-Hoover thermostat and semi-isotropic Parrinello–Rahman barostat^52^ with a time step of 0.5 and 5 ps, respectively. Both systems were run for 70 ns. For the first system, a temperature of 274 K and a pressure of 25 bar were used to promote hydrate growth. Whereas for the second system, the conditions were set to 350 K and 5 bar to promote hydrate dissociation.^11^

## Results and Discussion

### Comparison Between New and Existing Algorithms

We begin by comparing the performance of the individual order parameters discussed in the Order parameters section with that of the algorithm introduced in this section. In Figure 4, we compare the results obtained when sI and sII hydrates are simulated (panels a and b, respectively). The results show that the order parameters commonly used in the literature identify the solid hydrate phases and the liquid phase after computing the local average. The distinctions are also often carried out by averaging within each window along the direction perpendicular to the solid–liquid interface (*z* direction of the simulation box). The transition regions (highlighted) can also be identified by locating the peak of the normalized derivative of the individual order parameters along the *z*-axis. The average thickness of the interfacial region, as obtained from the order parameters applied to the sI system, is 4.0, 3.9, and 4.0 Å, respectively. At the same time, those of the sII systems are 4.2, 4.05, and 4.1 Å.

**Figure 4:**
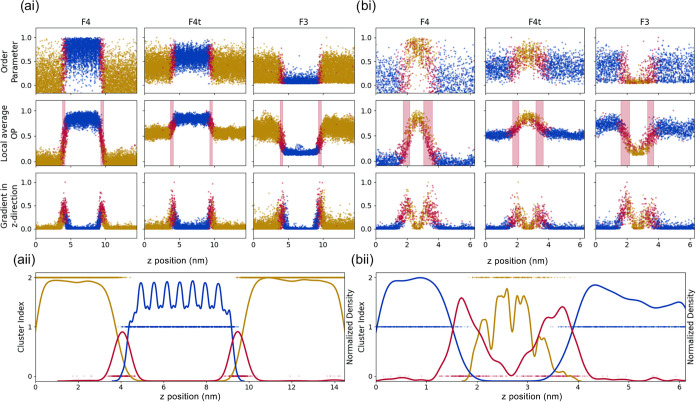
Comparison of F3, F4, and F4t order parameters (OPs) with clustering results for (a) an sI hydrate system at 50 ns and (b) an sII hydrate system at 18 ns. The top panels (ai and bi) display the raw order parameter values, their local averages within the first coordination shell, and their gradients along the *z*-direction. Highlighted regions in the local average plots indicate the interfacial layer between the hydrate and liquid phases. Each data point corresponds to a single water molecule. The bottom panels (aii and bii) show the output of the clustering algorithm. Cluster indices are assigned arbitrarily and used as *y*-axis coordinates. Notably, the interfacial region is consistently classified into a distinct cluster (red). Normalized spatial density distributions for each cluster are also provided to illustrate their structural localization.

While these results are overall satisfactory, we note that identification is only feasible on a global scale. The dispersions in all cases show that a significant number of false positives are routinely identified in both liquid and solid structures, and that the order parameters do not allow us to distinguish between different hydrate structures (i.e., results obtained for sI are similar to those for sII structures). All these uncertainties make the identification of the interfacial region fuzzy at best.

By comparison, we report the results obtained when the new algorithm is implemented in Figure 4aii,bii. A clear separation is observed between the hydrate and liquid clusters. Although the interfacial cluster appears somewhat less distinct, with occasional false positives, the normalized density curve effectively refines the identification of the interfacial region by isolating it around the highest peak. The new methodology offers significant advantages over traditional order parameters, particularly in terms of accuracy and resolution. It can be seen that the statistical uncertainty in the proposed algorithm is significantly smaller as it differentiates between hydrate, liquid, and interfacial water. The thickness of the interfacial region obtained is also higher than that computed from the conventional order parameter. In the melting system, thickness has reached the nanometer scale, which is closer to the values reported in the literature.^29,34,53^

Furthermore, it can be observed from Figure 4bi that the order parameter results are ambiguous at the interface, particularly during melting. On the other hand, the clustering with the cavity-finder algorithm yields accurate differentiation among all states, even during melting. All these features confirm that the algorithm proposed here offers a comprehensive description of the interfacial properties of hydrate systems.

The cavity-finder algorithm can distinguish among different types of hydrate cages. However, when its output is used as an input to the density-based clustering algorithm, the data is intentionally simplified into binary states (i.e., the presence or absence of a cavity) to reduce complexity and noise in the clustering process. As a result, while the clustering output successfully differentiates between broader states such as liquid, hydrate, and interfacial water, it does not reflect the detailed structural information about specific hydrate cage types. This capability could, however, be achieved by using the cavity-finder algorithm in conjunction with density-based, unsupervised clustering.

We also benchmarked the proposed algorithm against GRADE by comparing their computational costs and classification accuracies across four hydrate systems (single-frame) with different guest compositions. System 1 consists of a fully occupied 3 × 3 × 3 sI hydrate containing 60% CO_2_ and 40% CH_4_. System 2 comprises an empty 3 × 3 × 3 sH hydrate slab. System 3 is a 3 × 3 × 3 sII hydrate with THF occupying the large cages, while System 4 consists of a 2 × 2 × 2 sII hydrate in which THF occupies the large cages, and CO_2_ occupies the small cages.

The classification accuracy for both methods was evaluated using false-positive and false-negative rates, and the results are summarized in Figure 5. Overall, the cavity-finder approach exhibits higher accuracy than GRADE across all four systems, achieving 100% accuracy in Systems 1 and 4. Occasional false positives were observed with the cavity-finder approach, in which bulk water molecules were misclassified as hydrate cages, as shown in System 3. However, such instances are rare, yielding a high overall accuracy of 98%. Notably, the cavity-finder approach also successfully identifies sH hydrates with an accuracy of 87%, as shown in Figure 5 panel (b).

**Figure 5:**
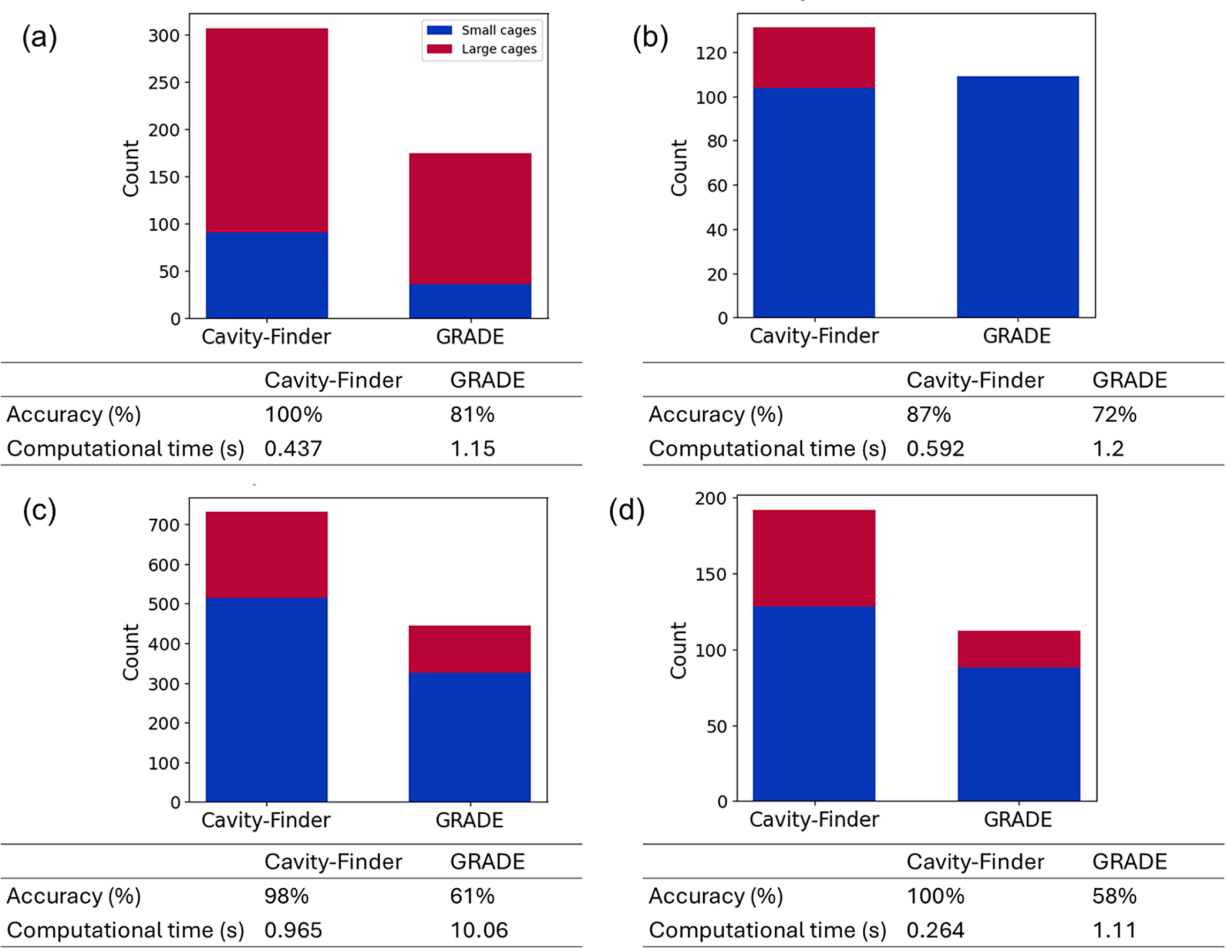
Performance comparison between cavity-finder and GRADE algorithm in terms of accuracy and computational cost for (a) sI hydrate system containing 60% CO_2_ and 40% CH_4_, (b) sH hydrate system, (c) sII hydrate system containing THF, and (d) sII hydrate system containing THF and CO_2_.

In contrast, while GRADE shows no false positives in any of the four systems, it exhibits a significantly higher false-negative rate, leading to lower overall accuracy. In addition, the cavity-finder approach incurs substantially lower computational cost than GRADE across all systems considered. Comparison of longer trajectories will also be discussed below.

### sI Hydrates Growth

As illustrated in Figure 4, the unsupervised algorithm applied to the growing sI CH_4_ hydrate clustered the water molecules into three structures: cluster 0, cluster 1, and cluster 2. Figure 6 displays VMD snapshots of the hydrate system, highlighting distinct clusters of molecules, each colored differently to facilitate visualization. From the snapshot, it becomes apparent that cluster 1 is a hydrate-like molecule. Cluster 0 represents the interfacial layer between the hydrate and liquid water, while liquid water is represented by Cluster 2. As such, we can infer that this algorithm can identify the states of each molecule.

**Figure 6:**
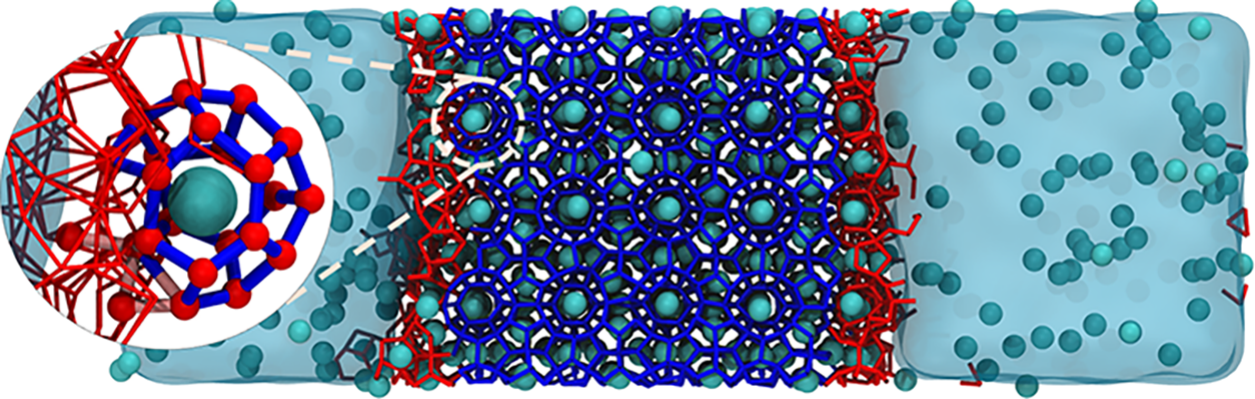
Clustering results from the sI hydrate system at the end of the simulation (i.e., 50 ns). The cyan spheres represent Carbon Dioxide molecules. The dark blue sticks illustrate the hydrogen bonds that form the hydrate lattice. Red sticks represent the interfacial QLL between the hydrate and the liquid. A zoomed-in snapshot of the interface is depicted on the right. The red spheres and pink sticks in the zoomed-in snapshot represent the oxygen atoms of water and the semicage structures present within the interfacial layer.

Given the structural similarities between hydrates and ice, we adopt the quasi-liquid layer (QLL) concept from ice literature because it has been widely proposed that hydrate surfaces exhibit a liquid-like layer analogous to that found on ice.^54^ Hence, we interpret the interfacial layer identified in the clustering results as the QLL. The QLL thickness attained at the end of the simulation (i.e., 50 ns of simulation) is about 8 Å thick on both interfaces. Zooming into the QLL as illustrated in Figure 6, we observe a layer of semicages (depicted in pink) and an additional layer of water molecules. The semicage layer primarily consists of ring structures resembling fully formed hydrate frameworks; however, these semicages exhibit distortions or are incomplete. The inclusion of both semicages and water in the identification of the interface is likely to yield a QLL thickness higher than that obtained by the order parameters alone.

For completeness, we note that the QLL thickness estimated here is thinner than some values reported in the literature.^34,35^ This discrepancy happens because the sI hydrate simulated in Figure 6 is undergoing growth rather than dissociation. Dissociation is, in fact, associated with thicker QLLs.^33^ The ability to distinguish between these two phenomena further reinforces the reliability of the new proposed algorithm.

We also compared the performance of our cavity-finder with GRADE, as shown in Figure 7. Overall, GRADE detects fewer cages than cavity-finder. This observation is consistent with our previous benchmarking, which demonstrated that cavity-finder significantly reduces false negatives, albeit with a slightly higher risk of false positives. In addition, cavity-finder is substantially more computationally efficient, requiring about one-fourth of the time GRADE requires for a similar analysis. To substantiate this comparative performance, both methodologies were tested on five independent simulated trajectories, each consisting of 10 frames sliced from the growth simulation. Under these conditions, GRADE required an average computational time of 91.8 s, whereas the cavity-finder method required 19.8 s to perform the same analysis. This suggests a significant improvement in computational efficiency, although the performance of both methods is expected to depend on system size, trajectory length, and additional parameters.

**Figure 7:**
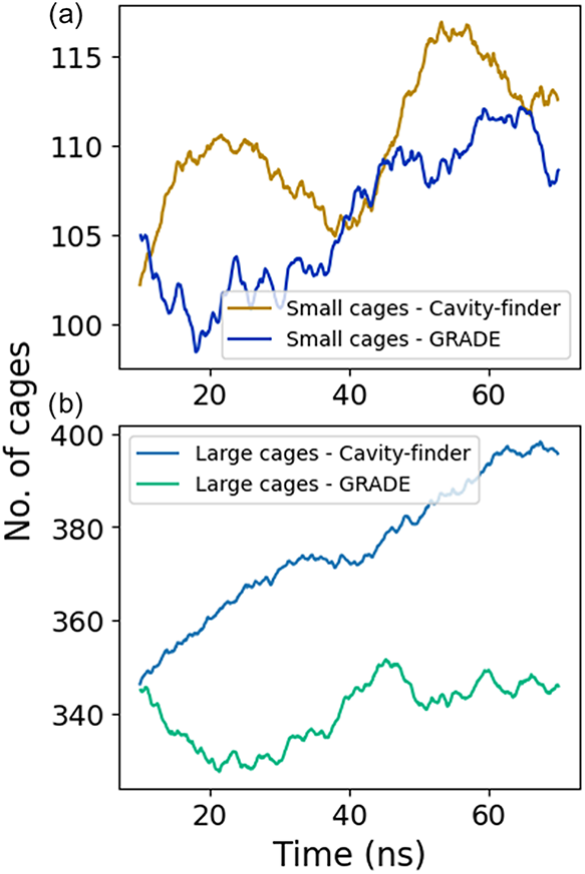
Comparison between Cavity-finder and GRADE algorithm in cage detection of (a) small cages, and (b) large cages, during sI hydrate growth.

### sII Hydrates Dissolution

The algorithm is also tested on a more complex hydrate dissociation system. Figure 8 shows the evolution profile of the dissociation process of the sII mixed CH_4_/Dioxane hydrate. It can be observed from Figure 8b that the cavity-finder algorithm is effective in identifying the hydrate cages. Beyond merely detecting the cages, the algorithm also effectively distinguishes between structural features, such as small and large cavities. We expect that the new algorithm would also be able to detect rare hydrate structures, provided they consist of 4-membered to 7-membered rings. The gap between the occupied and total identified cages indicates that the algorithm also successfully identifies empty cages. As the hydrate dissociates, this gap gradually narrows as empty cages melt, increasing the percentage of occupied cages. These features are not available when the order parameter algorithms are implemented alone, highlighting the ability of the newly proposed algorithm to distinguish not only between liquid, hydrate, and interface phases but also between different structures within the solid hydrate structure. We again compared the performance of our method with GRADE, as shown in Figure 9. The results are consistent with previous analyses, in which GRADE detects fewer cages than the cavity-finder.

**Figure 8:**
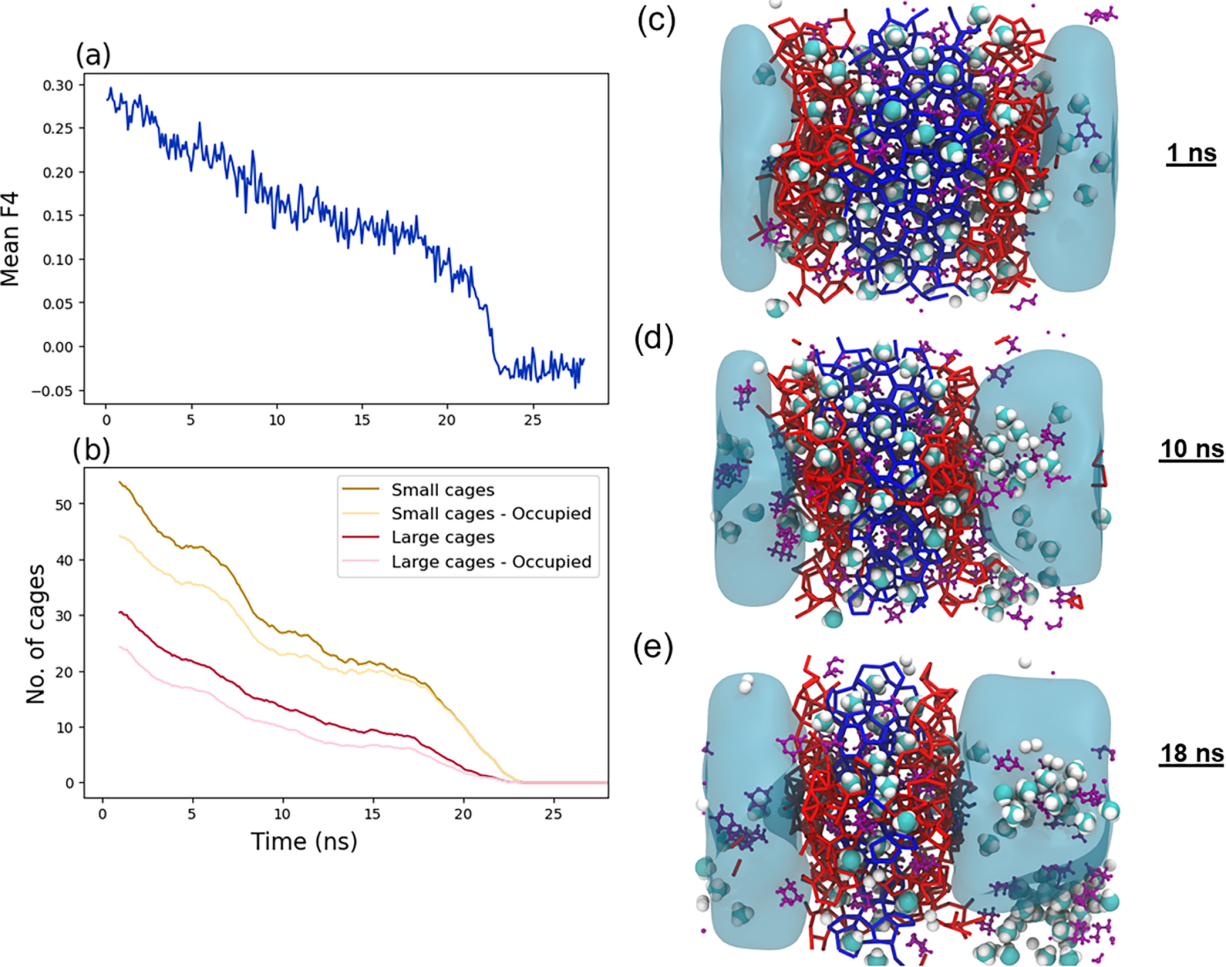
Evolution of the dissociation of sII hydrate. (a) illustrates the change of hydrate size using the *F*4 order parameter. (b) shows the change in the number of cages using the cavity-finder algorithm only. (c), (d), (e) are the VMD snapshots of the clustering result of a sII CH_4_/Dioxane hydrate system at 1, 10, and 18 ns. The red layers represent the interfacial cluster.

**Figure 9:**
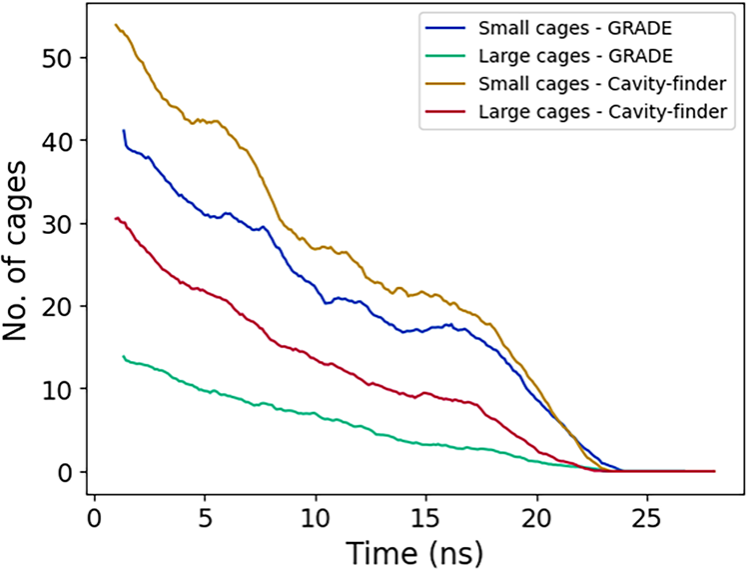
Comparison between the Cavity-finder and GRADE algorithms in cage detection during the dissolution of an sII Hydrate.

To visualize the results, we present color-coded simulation snapshots in Figure 8, panels c, d, and e. The thickness of the QLL at 18 ns (Figure 8e) is larger than 1 nm thick on both the left and right interfaces. As already stated, these observations are realistic both in the fact that the QLL are semiordered,^55,56^ and in the fact that the experimentally determined thickness is of the order of the nanometer scale.^29,34,53^ Furthermore, Figure 8d,e reveal that the interface becomes increasingly nonuniform as the hydrate undergoes dissociation, as shown in Figure 4b. We note that, also in this case, the cavity-finder-based algorithm remains robust, providing an accurate and consistent description of the interfacial layer. This suggests that the algorithm could be used effectively in investigating the growth/dissociation of 3D hydrate particles, such as those simulated at complex interfaces.

## Conclusions

Despite the widespread use of conventional order parameters, they fail to capture the full complexity of hydrate systems, particularly at the solid/liquid interface. To address this limitation, we introduce a comprehensive methodology that combines three traditional descriptors, *F*3, *F*4, and *F*4*t*, with a newly developed cavity-finder algorithm. These four features, together with the gradient of *F*4, define a five-dimensional space, which is then used as input to density-based DBSCAN clustering. This approach enables quick and accurate identification of distinct hydrate states, including liquid, solid, and interfacial phases. Most importantly, it enables detailed characterization of the quasi-liquid layer (QLL), a structurally complex region that is notoriously difficult to resolve with conventional methods. By integrating global order parameters with local structural insight, this framework provides a more complete and reliable understanding of hydrate behavior at the molecular level. The newly developed cavity-finder algorithm was also tested across CO_2_ sI growing and more complex mixed CH_4_-Dioxane sII melting hydrate systems, confirming its capability to accurately identify various hydrate structures, such as 5^12^, 5^12^6^2^, and 5^12^6^4^.^57^ The algorithm also effectively detects the guest molecules within each cage, including empty cages, offering a more comprehensive approach to analyzing hydrate formation and dissociation. This level of resolution is not achievable with the order-parameter approaches commonly used in the literature. It is anticipated that the new clustering algorithm can be modified to describe different structures by including additional order parameters as necessary.

## Data Availability

A Python implementation of the clustering algorithm presented in this paper is available at https://github.com/mme-ucl/clathrate_hydrate_classification.git.
